# Supplementary material for: A bioelectronic device for electric field treatment of wounds reduces inflammation in an in vivo mouse model
Source: PLoS One. 2024 Jun 14;19(6):e0303692. doi: 10.1371/journal.pone.0303692 (PMC11178234; doi:10.1371/journal.pone.0303692)
Supplement: S1 File — Contains S1 Table. Electric field device quality control check on 4.9.22, S2 Table. Electric field delivery experiment on 4.10.22–4.14.22, S3 Table. Test animal identification and biocompatibility evaluation, S1 Fig. Three iterations of microcontroller design, S2 Fig. Testing version 2 device current connection of electric field treatment using multimeters, S3 Fig. Bioelectronic device circuit system, S4 Fig. Re-epithelialization analysis, S5 Fig. Electrical stimulation modulated macrophage percentages during wound healing in vivo, S6 Fig. Experiment 20—High dose electric field wound treatment via device. (DOCX) [file pone.0303692.s001.docx]

**Supporting Information**

**Device Iteration**

The electric field microcontroller went through various iterations before our team decided on the final version to use for most of the experiments (S1 Fig.). Version 1 uses an external power supply and applies a voltage while recording the current. However, it can only be used while the mouse is under anesthesia. Version 2 is powered by a battery and has a programmed actuation cycle that resets every 24 hours. However, it does not record data. Version 3 is powered by a battery and has a programmed actuation cycle that resets every 24 hours. It does record data. Our team selected version 2 and that microcontroller was integrated into our device and used during our in vivo experiments.

Version 1


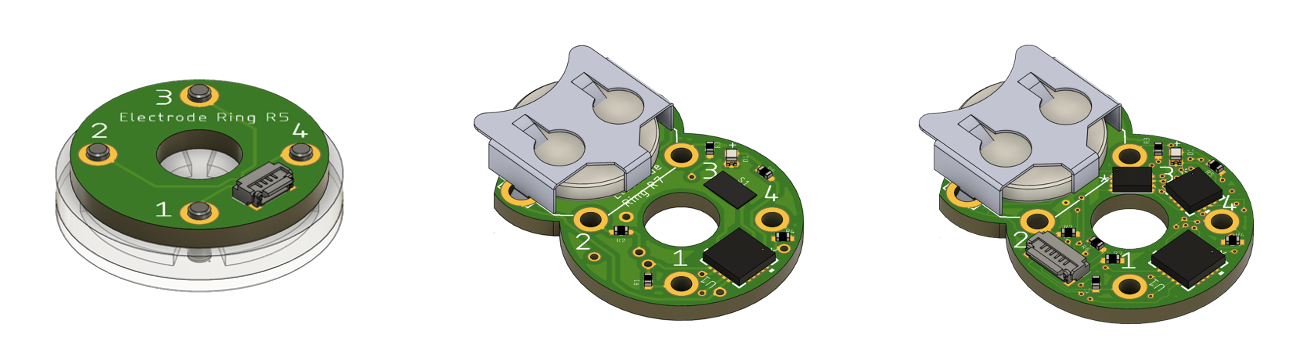


Version 2

Version 3

**S1 Figure. Three iterations of microcontroller design.**

To test current connection of version 1 device during in vivo experiment, we used three multimeters. The mouse had to be under anesthesia to actuate the EF treatment, which only lasted for 10 minutes (S2 Fig.).

**Quality Control**

We performed quality control using a digital multimeter on all fabricated devices. The devices that had all four channels working were selected to be used in the in vivo experiments (S1 Table). The testing method will be further explained in the following supporting information.

**S1 Table. Electric field device quality control check on 4.9.22.**

| Device | Channel 1 | Channel 2 | Channel 3 | Channel 4 | Pass/Fail |
| --- | --- | --- | --- | --- | --- |
| 1 | 0.034 V | 0.012 V | 0.024 V | 0 V | Fail |
| 2 | 0.063 V | 0.082 V | 0.003 V | 0.037 | Pass |
| 3 | 0.010 V | 0.041 V | 0.095 V | 0.030 V | Pass |
| 4 | 0 V | 0.042 V | 0.059 V | 0.068V | Fail |
| 5 | 0.041 V | 0.041 V | 0.081 V | 0.045 V | Pass |
| 6 | 0.018 V | 0.033 V | 0.026 V | 0.053 V | Pass |
| 7 | 0.053 V | 0.039 V | 0.022 V | 0.060 V | Pass |

**Testing Device Connection During In Vivo Experiments**

To test current connection during in vivo experiment for version 2 device, we used a digital multimeter (S2 Fig.). The positive (red) lead of the multimeter touches the hole of the resistor closest to the working electrodes. The negative (black) lead touches the soldered pin. This provides our team with a positive current reading that indicates the device is contacting the mouse wound and actuating the electric field treatment.


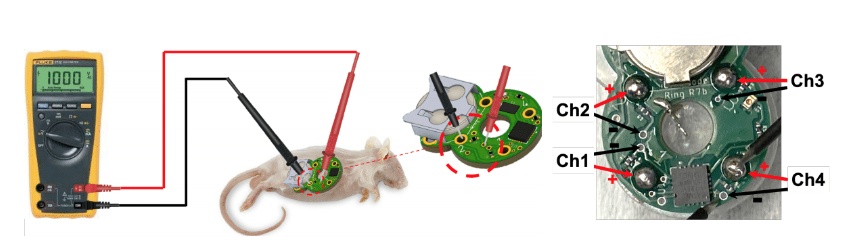

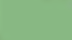


**S2 Figure. Testing version 2 device current connection of electric field treatment using multimeters.**

Please note, to stay within the healthy anesthesia dosage time for mice allowed we only took current measurements for channel 2 of our device during in vivo. We measured the current starting from day 0 until day 2 (S2 Table). On average the current was calculated to be ~10 μA for our various experiments we did throughout the year.

**S2 Table. Electric field delivery experiment on 4.10.22-4.14.22.**

|  |  | **Day 0 [3V]** | **Day 1 [3V]** | **Day 2 [3V]** | **Day 3** |
| --- | --- | --- | --- | --- | --- |
| **Device** | **Test Subject** | **Current** | **Current** | **Current** | **Harvest Tissue** |
| 3 | EF Delivery #1 | 4.7 μA | 12 μA | 8.6 μA | ✓ |
| 5 | EF Delivery #2 | 9.5 μA | 11.5 μA | 9 μA | ✓ |
| 6 | EF Delivery #3 | 17.6 μA | 10.5 μA | 8.7 μA | ✓ |
| 7 | EF Delivery #4 | 10.4 μA | 8.1 μA | 9.2 μA | ✓ |

**Calculating Electric Field**

The electric field strength at a wound site can be calculated using different formulas depending on the method of electric field generation. For our bioelectronic bandage that utilizes an ion pump electric field delivery unit, the electric field strength can be calculated using the formula E = V / d, where E represents the electric field strength in volts per meter (V/m), V represents the voltage in volts (V), and d represents the distance between the electrodes in meters (m). These calculations are crucial for understanding the electric field strength at a wound site and its potential effects on wound healing.


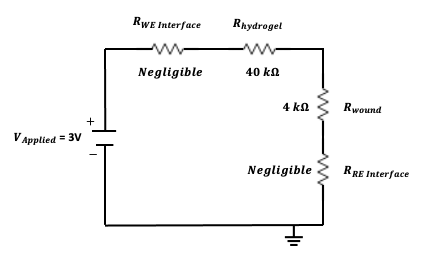


**S3 Figure. Bioelectronic device circuit system.**

Initially we assumed our EF strength was affected by resistance generated from the Ag/AgCl electrodes ($R_{WE Interface}$), hydrogel filled capillaries ($R_{hydrogel}$), wound resistance ($R_{wound}$), and Ag electrode ($R_{RE Interface}$) resulting in a voltage drop across the circuit system (S3 Fig.). However, after calculations we deemed the resistance generated from $R_{WE Interface}$ and $R_{RE Interface}$ to be negligible.

**Voltage Drop Calculation:**

$$V_{wound}= Voltage Drop$$

$$V_{wound}= \frac{R_{wound}}{R_{total}} \times V_{appiled}= \frac{4 k\Omega}{40 k\Omega}\times3V=0.3V$$

$$V_{wound}=0.3V$$

**Electric Field Calculation:**

$$d_{dbe}:distance between electrodes$$

$$E= \frac{V_{wound}}{d_{dbe}}= \frac{0.3V}{1.5mm} \times1000=200 \frac{mV}{mm}$$

$$E= 200 \frac{mV}{mm}$$

**DB-705: Biocompatibility Test**

**Objective:** This study was to evaluate the biocompatibility of novel bioelectronic devices placed subcutaneously in a rabbit model.

**Methods:** The study population consisted of 6 animals, (3 males and 3 females). There was one (1) control group and two (2) test groups. Each animal was scheduled to receive 12 implants: six (6) on the right and six (6) on the left dorsal region. Each animal (with the exception of Animal No. 4M) was implanted according to the Study Design Table.

Each animal was subjected to the surgical procedure described in Study Protocol DB-705, Section 14.7. Animal No. 4M (male in Group 1) did not tolerate anesthesia and died. After surgery, the other animals were allowed to recover and observed for the survival period of 30±2 Days. After completion of the survival period, the animals were euthanized; all implant sites were harvested, preserved in 10% NBF, and referred for histopathology.

From each animal, representative samples of brain (5), heart, lung (2), liver, kidneys (2), testicles (2) / ovaries (2), mesenteric lymph node, adrenal glands, spleen, thymus, quadriceps (2), and eyes (2) were obtained, for a total of 21 samples. These samples were fixed and preserved in 10% NBF and referred for histopathology.

**Results:** During sedation/anesthesia and prior to implant procedure, Animal No. 4M did not tolerate the anesthesia and died. The remaining five (5) animals were successfully implanted, recovered from surgery, and survived to Day 29. There were no adverse events in any of the five (5) animals.

Body Weight: The individual animal body weight and body condition scores were stable from treatment surgery through Day 29.

Clinical Pathology: The baseline and Day 29 complete blood counts for all 5 rabbits on study were normal. The clinical chemistry values remained normal for all animals at baseline and Day 29 except for CPK (creatine phosphokinase). This enzyme becomes elevated after intramuscular (IM) injections. The animals were sedated with an IM injection at both baseline and Day 29 resulting in CPK elevations. This elevation was not related to the implanted devices. All rabbits remained healthy throughout the in-life period.

Study Specific Observations: All 5 animals recovered well post-operatively. During the survival period, there were some minor findings including: on Day 1 postoperatively, all 5 animals exhibited incisions that were intact with some dried blood around wound. This resoled by Day 2 with exception of Animal No. 5M (Group 2). Animal No. 5M wound sites were stained with blood (Day 3 to Day 4) and then scabbed until Day 12. No intervention was necessary, and the animal successfully completed the study.

**Conclusion:**

The study objectives were met.

The rabbits did not exhibit any test article-related abnormalities throughout the study. All 5 animals successfully underwent the surgical procedures, recovered, and completed the 29-day survival period. At the completion of the study, all animals were evaluated as being in optimum health.

**S3 Table. Test animal identification and biocompatibility evaluation.**

| Group ID | Animals ID / Sex | Testing Samples | Biocompatibility Evaluation |
| --- | --- | --- | --- |
| 1 | ~~4M~~ | ~~Control Article: PDMS + Parylene-C~~ | The ISO 10993-6:2016 Ranked Reactivity scores for Test Article 1 and Test Article 2 implants relative to the Control Article implant were classified as **minimal to no reaction** in the subcutaneous rabbit model at 29 days. No pathologic changes indicative of toxic injury were found in the non-target organs evaluated in any of the 5 animals. |
|  | 1F | Control Article: PDMS + Parylene-C |  |
| 2 | 5M | Test Article 1: PDMS + Parylene-C + Capillary Tube |  |
|  | 2F | Test Article 1: PDMS + Parylene-C + Capillary Tube |  |
| 3 | 6M | Control Article: PDMS + Parylene-C + Capillary Tube + Hydrogel |  |
|  | 3F | Control Article: PDMS + Parylene-C + Capillary Tube + Hydrogel |  |

**Closer Look at Histology Images Indicating New-Epithelial Layers**

**
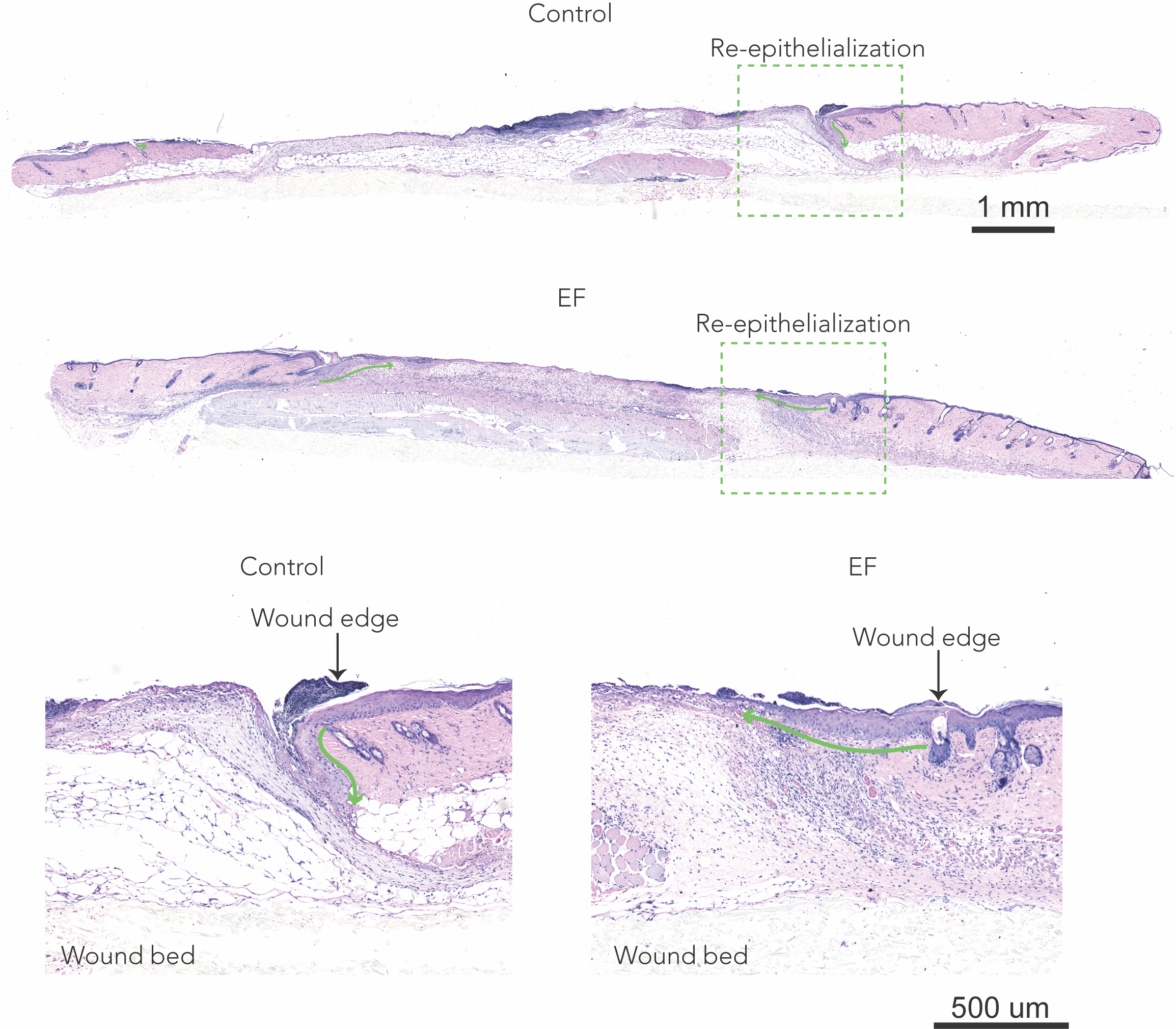
**

**S4 Figure. Re-epithelialization analysis.**

**M1 and M2 Percentages**

**
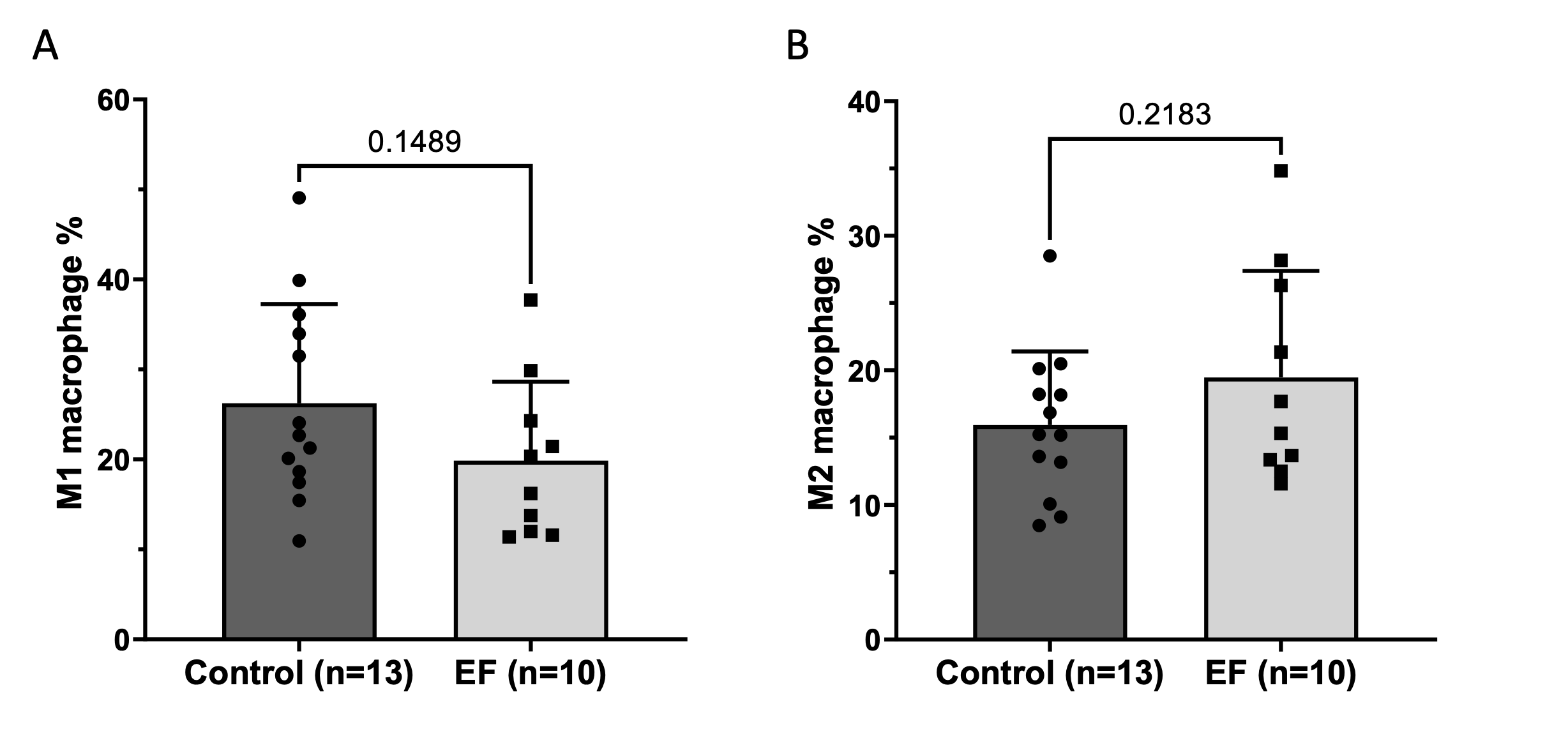
**

**S5 Figure. Electrical stimulation modulated macrophage percentages during wound healing *in vivo*.**

**M2 Marker: Control Versus Electric Field Treatment**


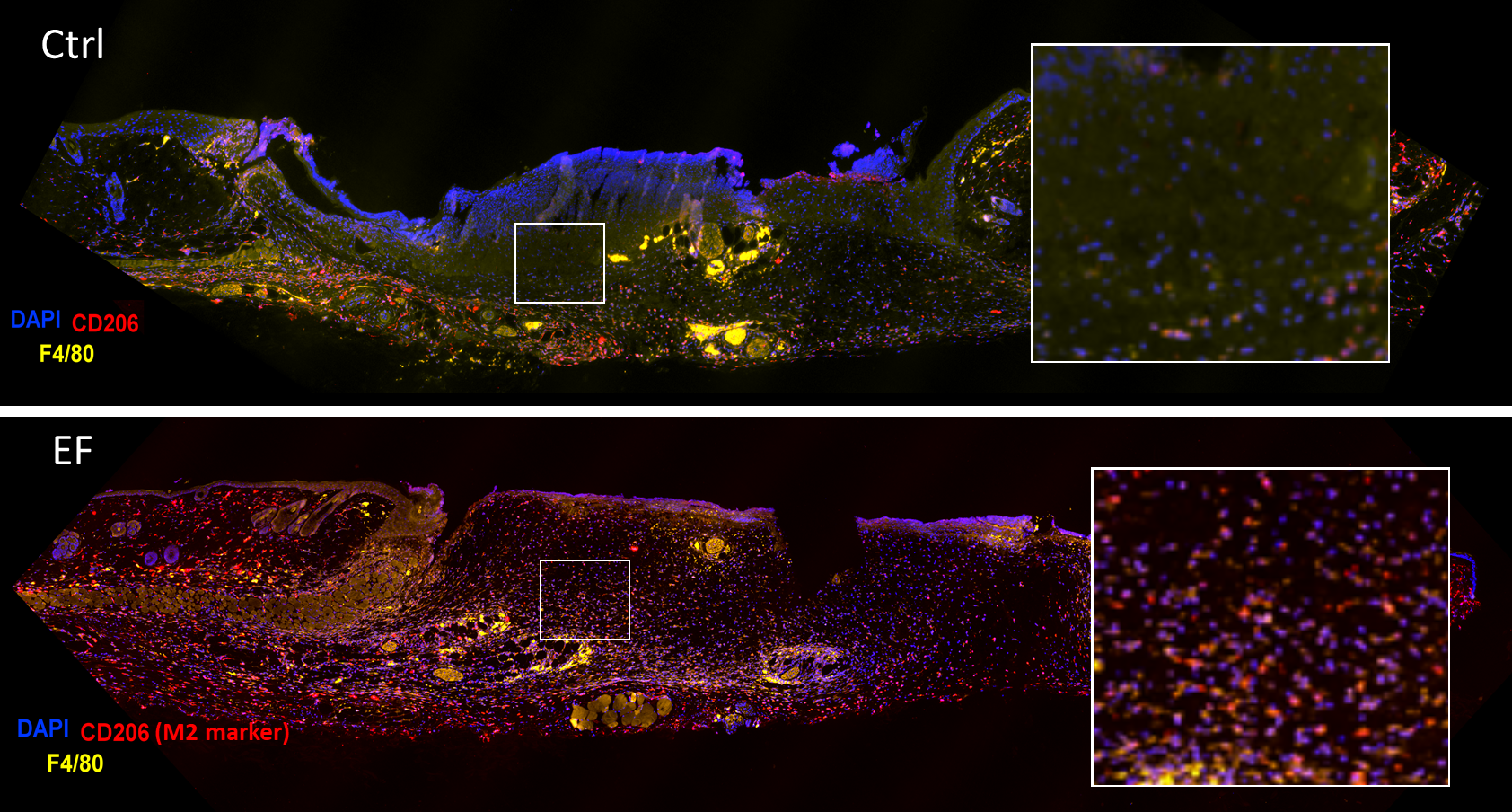


**S6 Figure. Experiment 20 - High dose electric field wound treatment via device.**
