# Supplementary material for: A bioelectronic device for electric field treatment of wounds reduces inflammation in an in vivo mouse model
Source: PLoS One. 2024 Jun 14;19(6):e0303692. doi: 10.1371/journal.pone.0303692 (PMC11178234; doi:10.1371/journal.pone.0303692)
Supplement: Attachment — Submitted filename: 10_2023_Response to Reviewer.docx [file pone.0303692.s002.docx]

Paper No.: # PONE-D-23-22607

Paper Title: A Bioelectronic Device for Treatment Of Wounds Improves Outcomes in An In Vivo Mouse Model

The manuscript has been revised according to the reviewer’s comments and we now believe the manuscript is ready for publication.

Reviewer #1

In this work, the authors present a bioelectronic device for assisting in the treatment of wounds that possesses wearable and programmable properties. The bioelectronic device achieves modulation of wound regenerative cell migration and macrophage phenotype and differentiation by applying a programmable electric field to a mouse wound, leading to wound re-epithelialization and healing. This wearable electronic device proposed by the authors not only supports the application of programmed applied electric fields for in vivo wound healing, but also provides new ideas and a basis for the development of further techniques based on the modulation of macrophages and inflammation for better wound healing. The content of this work is interesting and deserves to be studied in depth. However, I have some concerns about the current version of this work.

| 1 | Comment | Generally speaking, the wound surface has irregularity, and sewing often destroys part of the wound surface in vivo, which will easily cause the device to cause secondary damage to the wound. In addition, skin wounds tend to exude tissue fluid at the wound surface, and the effect of tissue fluid on the bioelectronic device is also considered and eliminated during biological experiments. |
| --- | --- | --- |
|  | Response | Mice skin is not attached firmly to the muscle fascia layer underneath. Instead of stretching and destroying the wound tissue, the sutures helped the wound opening stay at the same location where the device rests on, rather than sliding around when the animal moved. The device is held in place by adhering it to the skin using Tegaderm tape. The splints are the only items sewn in place. However, the secondary damage described above is not a concern because splinting the mouse wound allows the repair process to be dependent on epithelialization, cellular proliferation, and angiogenesis, which closely mirror the biological processes of human wound healing. Therefore, the silicon sutures were sewn accordingly with the standard pre-approved protocol (Dunn, L. et al., Murine Model of Wound Healing. J. Vis. Exp. (75), e50265, doi:10.3791/50265 (2013).).  We did not observe much wound exudates in the current mouse model for the short-term experiments but agree that when moving forward to longer experiments or a large animal model, the effect of the device conductivities with wound fluid needs to be considered.  To prevent any possible confusion to our audience, we will be adding a few sentences to the section Animal Wounding and Daily Treatment on page 8.  “Splinting the mouse wound allows the repair process to be dependent on epithelialization, cellular proliferation, and angiogenesis. The sutured splints cause no secondary damage to the wound bed because the skin is not attached firmly to the muscle layer underneath. Instead, it stabilizes the wound and provides a platform to maintain the bioelectronic device localized to the wound site. The bioelectronic device or a mock PDMS control device rest on top of the splint rings allowing only the actuating bottom surface of the device to come in contact with the wound bed. Tegaderm dressing (3M, Maplewood, MN) maintains the device stationary to the wound preventing any slippage that can result in treatment failure.” |

| 2 | Comment | In this bioelectronic device, hydrogel is applied as the interface material between the device and the wound, and the solubilizing absorption and enrichment effect of hydrogel also promotes wound healing, does this have a superimposed effect with the wound healing effect of the bioelectronic device? If the stimulatory effect of an applied electric field on wound healing is explored, it is suggested that a control group where the electronic device is installed but no electric field is applied could be added for analysis. |
| --- | --- | --- |
|  | Response | The hydrogel-filled capillaries establishes direct contact between the device and the wound bed for efficient bioelectronic transmission of the electric field. The volume of the hydrogel including all four capillaries equates to approximately 0.157 μL. The volume used in the device is negligible. Thus, it is unlikely that there would be a hydrogel induced enrichment effect.  To prevent any possible confusion to our audience, we will be adding a few sentences to the section Results and Discussion on page 4.  “All materials that come in contact with the wound resulted in no adverse effect during biocompatibility testing. For example, the volume of the hydrogel including all four capillaries equates to approximately 0.157 μL. The volume used in the device is negligible thus, rendering hydrogel unlikely to induce toxicity or an enrichment effect that can aid in healing. “ |

| 3 | Comment | The selected experimental items and control data are small, and the treatment test with three days as the cycle lacks certain rigor and persuasiveness, the results of a longer cycle can be taken for observation and analysis. At the same time, the healing effect of mouse wounds should be tested by providing some data on the change of wound size, not only the wound re-epithelialization rate and macrophage phenotype percentage of the wound, so as to intuitively correspond to the incentive effect of wounds. |
| --- | --- | --- |
|  | Response | We appreciate the suggestion and agree that longer observation period should be performed for wound healing. However, designing miniature devices for mouse use is not an easy task. The function of the device was limited by the overall size and the chip inside. We also required to pay extensive attention to prevent blocking animal’s movement when the device was installed. Thus, we only report the proof-of-concept results in this manuscript with short-term experiments but will design long-term experiments when moving forward to a large animal model.  We note however, that histological assessment of wound epithelialization is a more definitive measurement of healing than evaluating the wound surface. That is because 1) the new epithelial tongue is just a few cells deep and cannot be visualized macroscopically, and 2) the wound edges on a surface image are often obscured by eschar, or by the inability to focus the different planes of the wound edge simultaneously sharply on a living animal’s dorsum that is curved. As noted above, this 3-day experiment is proof-of-concept that the device improves 2 classically evaluated outcomes of healing: wound re-epithelialization and macrophage polarization.  “We decided to focus our analysis on re-epithelialization and macrophage phenotype. Histological assessment of wound epithelialization is a more definitive measurement of healing compared to evaluating the wound surface because the new epithelial tongue is just a few cells deep and cannot be visualized macroscopically. Surface images of wound edges are often obscured by eschar or the inability to focus sharply on different planes simultaneously on a living animal’s curved dorsum.” |

| 4 | Comment | What is the basis for choosing an applied electric field of 125 ± 75 mv/mm for the electronic device? Some prospective analysis can be made of the effect of the parameters associated with this bioelectronic device on wound healing in vivo and the optimization of the associated parameters. |
| --- | --- | --- |
|  | Response | Independent laboratories using different technologies have demonstrated that the endogenous electric fields at skin and cornea wounds of rodents, human, and other mammals typically range from 100 to 200 mV/mm. Those include glass microelectrode, microneedle arrays, and bioelectrical imager (Barker et al., Am J Physiol. 1982;242(3): R358-66. Mukerjee et al., Conf Proc IEEE Eng Med Biol Soc. 2006:2006:4326-8. Nuccitelli et al., Wound Repair Regen. 2011;19(5):645-55). Those measurements have been corroborated with electric current measurements using a non-invasive vibrating probe (Zhao et al., Nature. 2006;442(7101):457-60. Reid et al., Nat Protoc. 2007;2(3):661-9).    The chosen electric field of 125 ± 75 mV/mm for the electronic wound healing device is based on the above measurements and taking consideration of effectiveness and safety. We aim to enhance the electric field at a skin wound to a range that has been proven to be biologically compatible and conducive to promoting directional cell migration and wound healing without causing harm to cells and tissues.    Prospective analysis is a great suggestion. We are working on that. When the parameters associated with this bioelectronic device can be accurately modulated and exerted on the tissues in the wounds, we will carry out a prospective study and analysis.  To prevent any possible confusion to our audience, we will be adding the following sentences to the section Results and Discussion on page 3:  “We designed our bioelectronic device to emit an electric field strength of 125 ± 75 mV/m because prior studies indicate that the endogenous electric fields at skin and cornea wounds of rodents typically range from 100 to 200 mV/m [29]. We aim to enhance the electric field at a skin wound to a range that has been proven to be biologically compatible and conducive to promoting directional cell migration and wound healing without causing harm to cells and tissues.” |

| 5 | Comment | Some of the pictures are not clearly drawn and contain logical errors. The drawing of Figure 2C is inconsistent with the contextual description, and the picture of electronic equipment in Figure S2 in the supporting information does not match the previous discussion. Please check carefully. |
| --- | --- | --- |
|  | Response | We appreciate your comment. We were able to see the inconsistency where we mention 250-minute treatment time versus the 6-hour treatment that was mentioned in the figure 2C description and is shown in the image itself. The correct time is 250-minutes so we will make that adjustment to the figure and its description. We were able to see we needed to make an update to figure S2 from supplementary information. The image shows version 3 of the PCB, but we decided to go with version 2 for our in vivo experiments. I have made the proper edits to the image to match the electronic equipment.  During the review of all our figures, I found an issue that needed to be fixed in Figure 3B. The re-epi image for electric field application had inconsistent zoomed in cropped sections that needed to be updated. This was fixed and updated in the final manuscript. |

| 6 | Comment | The format of some references is not standardized. |
| --- | --- | --- |
|  | Response | I have updated the reference format to match Plos One standard. |
