# Supplementary material for: A bioelectronic device for electric field treatment of wounds reduces inflammation in an in vivo mouse model
Source: PLoS One. 2024 Jun 14;19(6):e0303692. doi: 10.1371/journal.pone.0303692 (PMC11178234; doi:10.1371/journal.pone.0303692)
Supplement: Attachment — Submitted filename: 01_2024_Response to Reviewer.docx [file pone.0303692.s003.docx]

Paper No.: # PONE-D-23-22607

Paper Title: A Bioelectronic Device for Electric Field Treatment of Wounds Improves Outcomes in An In Vivo Mouse Model

The manuscript has been revised according to the reviewer’s comments and we now believe the manuscript is ready for publication.

Reviewer #2: The authors have thoroughly revised the manuscript, and the manuscript is now on the sharp for publication.

Reviewer #3: Hernandez et al. extended their previous study (Ref#28) for a proof-of-principle application of the electric field (EF) treatment on wound healing in an in-vivo setting. For this, they used EF for 3 days to a surgical wound. Although conceptually novel and interesting, the data lack technical and methodological robustness (see below). Moreover, the authors did not perform any new experiments to address the concerns of the previous reviewer (comment#3), which were justified, relatively easy and feasible experiments. I have following comments:

| 1 | Comment | In line #112-114, authors claimed a decreased in M1 macrophage counts and unchanged M2 macrophage counts in treated mice. To this reviewer the representative images showed a decreased total DAPI+ cells in treated mice including F4/80+ macrophages, M1 like macrophages, but without any change in M2 like cells compared to untreated mice. So, I agree with reviewer 1 the need to quantify macrophage phenotype percentages in addition to the M1/M2 ratio. |
| --- | --- | --- |
|  | Response | We agree with your comment that the macrophage phenotype percentages are also important for evaluating the effects of electrical stimulation on wounds. Additional information is now included s follows:  “In the wounds treated with our EF device, the percentage of M1 macrophages was slightly reduced while that of the M2-like cells showed a tendency of increase when compared to the non-EF control (S4 Fig.). However, the M1/M2 ratio was significantly reduced by 30.6% after EF treatment (Fig. 3C).“  We have also included a new figure in supplementary information:    **S4 Figure. Electrical stimulation modulated Macrophage percentages during wound healing *in vivo*.**  Mean M1 (A) and M2 (B) percentages at the wounds from experiments shown in Figure 3 A and B. Total cell numbers were quantified based on the DAPI-stained cell nuclei. Data is expressed as mean ± SD. Student’s t-test. P values are as indicated. |

| 2 | Comment | In line # 118-119, authors claimed increased M2 macrophages due to proliferation which is contrary to the statement in line # 112-114. There is a need to quantify proliferation and apoptosis for both macrophage phenotypes before making such claim. |
| --- | --- | --- |
|  | Response | Thank you for pointing this out. Sorry for the confusion. We meant that EF stimulation promotes the “proliferation of macrophages”. So, we revised the sentence to the following:  The results suggest that EF stimulation to mouse wounds may be promoting the healing process by altering macrophage phenotypes, through the reduction of inflammatory M1 phenotype macrophages, thereby decreasing inflammatory stimuli and promoting earlier wound healing, such as rapid granulation and the accompanying tissue generation, by increasing the relative presence of reparative M2 macrophages.” |

| 3 | Comment | In line # 127-129, authors claimed significantly increased re-epithelialization with a p-value above >0.05 which is against the any significant value in statistical analysis. This is a very premature claim. Moreover, authors should provide higher magnification images with clearly visible new-epithelial layers. |
| --- | --- | --- |
|  | Response | We agree with your comment and changed the manuscript as follows:  “Moreover, the histomorphometric analysis of the treated and control wound beds indicated a trend towards and increase in wound edge re-epithelization (Fig. 3D).”  We have also provided higher magnification images of the new epithelial layers from Figure 3B in the supplementary information. The updated figures is as follows:  **Closer Look at Histology Images Indicating New-Epithelial Layers**    **S4 Figure. Re-epithelialization analysis.** Images of histology results and re-epithelialization marking the new re-generated layer. |

| 4 | Comment | Statistics. It is not clear which student’s t-test was used and the rationale for this. In addition, I did not find the reason to mention about ANOVA in the method part. Moreover, there is inconsistency in the biological replicates presented in figure 3 and in the figure legend. |
| --- | --- | --- |
|  | Response | We used unpaired 2-tailed student t-test. We have revised the manuscript with rationales, ANOVA has been removed from the statistics.  The updated manuscript reads as follows:  “All statistical data were analyzed using Prism 9 software, the mean with standard deviation or standard error of the mean was reported and comparisons were made using a 2-tailed un-paired Student's t-test because we compared the means of two groups, with the data sets following a normal distribution and have unknown variances.” |
