# Supplementary material for: A bioelectronic device for electric field treatment of wounds reduces inflammation in an in vivo mouse model
Source: PLoS One. 2024 Jun 14;19(6):e0303692. doi: 10.1371/journal.pone.0303692 (PMC11178234; doi:10.1371/journal.pone.0303692)
Supplement: Attachment — Submitted filename: 04_2024_Response to Reviewer.docx [file pone.0303692.s004.docx]

Paper No.: # PONE-D-23-22607

Paper Title: A Bioelectronic Device for Electric Field Treatment of Wounds Improves Outcomes in An In Vivo Mouse Model

The manuscript has been revised according to the reviewer’s comments and we now believe the manuscript is ready for publication.

Reviewer #4: In this paper, the authors successfully prepared a standalone, wearable, and programmable electronic device to administer a well-controlled exogenous EF, aiming to accelerate wound healing in a mouse model in vivo. However, there are some major concerns and questions the authors should consider before publication:

| 1 | Comment | In Figure 3D, the statistical result shows that the P-value above 0.05, then how to support the conclusion, this point seems to need to be explained. |
| --- | --- | --- |
|  | Response | We agree that the statistically significant response to the applied EF is the change in the macrophage phenotype and shift of M1/M2 ration (Figures 3a, 3c). Our conclusion is thus based on this significant finding, as noted: “The results suggest that EF stimulation to mouse wounds may be promoting the healing process by altering macrophage phenotypes, through the reduction of inflammatory M1 phenotype macrophages, thereby decreasing inflammatory stimuli and promoting earlier wound healing, such as a rapid granulation and the accompanying tissue generation, by increasing the relative presence of reparative M2 macrophages”  As the reviewer points out, the change in re-epithelialization was not significant at p value of 0.05, (likely due to inclusion of an outlier in the control group) and for that reason we indicate in the text that the change is trending rather than statistically significant: “the histomorphometric analysis of the treated and control wound beds indicated a trend towards an increase in wound edge re-epithelization” . We have also edited the abstract accordingly |

| 2 | Comment | In the in vivo part, why the sample number of different groups is inconsistent. |
| --- | --- | --- |
|  | Response | That is a correct observation. The control samples are taken from controls performed concomitantly with the reported EF experiments, as well as controls in other wound experiments thus the larger number of controls that EF wounds. The IHC analysis for M1/M2 could only be performed on histologic sections that had full dermal tissue visible in the section. Therefore, not every section/ wound analyzed for re-epithelialization could not be imaged by IHC for M1/M2, hence fewer samples for that analysis relative to the re-epithelialization analysis. |

| 3 | Comment | A good excellent academic paper should not only show the experimental results, but also give an in-depth discussion on these results. The author should pay attention to it. Some related studies (such as ACS Applied Materials & Interfaces 2022 14 (26), 29491-29505; Cell Mol Biol Lett. 2023 Jul 28;28(1):61, and 2024 Feb 5;29(1):24. can be cited and discussed to improve the discussion of this work. |
| --- | --- | --- |
|  | Response | I am sorry, these citations are not relevant. There are about peptides and chronic wounds and not Ef and acute wound. The irrelevance of these citations is reinforced by the fact that none of the work highlighted by the reviewer cites any papers on electric field treatment wound. This does not mean the work highlighted by the reviewer is not interesting, it is just not relevant here. We have upcoming publications that involve delivery of biochemicals to the wound and we will make sure to cite the excellent work that was brough to our attention. Cool stuff! |

Reviewer #5: This manuscript has novel findings, and it may benefit in treating wounds. However, there is a major concern that this manuscript is based on just one significant result, which is the M1/M2 macrophage ratio. The percent re-epithelialization results were not significant in control versus EF treatment. The study lacks other criteria that may support the conclusions. Below are the concerns in detail:

| 1 | Comment | The re-epithelialization were performed on H&E stained skin section which is not clear, however, the migrating keratinocytes can be better evaluated by keratin 14 staining which is the marker for keratinocytes or by keratin 17 staining as described in this publication  (<https://www.ncbi.nlm.nih.gov/pmc/articles/PMC4086220/> and <https://www.sciencedirect.com/science/article/pii/S0022202X15327469>). |
| --- | --- | --- |
|  | Response | We politely disagree with the reviewer and, at this stage (the paper has been under review for over 1 year), we simply do not have the resources nor the personnel to perform additional experiments because what the reviewer suggests, at the 3^rd^ round of review, was not in our initial protocol. However, we acknowledge the limitations of this work and we have edited the conclusions as follows  “ These are preliminary results and additional future studies using keratin 14 staining to evaluate keratinocites as well as other markers of wound healing such as pro-inflammatory cytokines either as immunohistochemistry or RNA or Western analysis of the skin samples would will be beneficial to confirm the conclusions of this work.  “  We would also like to add that based on the search results, there are several key advantages to using H&E staining to measure wound re-epithelialization:   1. H&E staining allows for direct visualization and quantification of the re-epithelialization process[1](https://journals.plos.org/plosone/article?id=10.1371%2Fjournal.pone.0018410)[3](https://journals.sagepub.com/doi/full/10.1080/01926230701584189). The search results show that researchers were able to measure the distance between the original wound edge and the leading edge of the re-epithelialized area using H&E stained tissue sections[1](https://journals.plos.org/plosone/article?id=10.1371%2Fjournal.pone.0018410). 2. H&E staining provides a comprehensive view of the wound healing process, including not just re-epithelialization but also other aspects like inflammation and granulation tissue formation[3](https://journals.sagepub.com/doi/full/10.1080/01926230701584189). This allows for a more holistic assessment of wound healing. 3. H&E staining is a well-established, widely used histological technique that is relatively simple and cost-effective compared to more specialized stains like keratin immunofluorescence[1](https://journals.plos.org/plosone/article?id=10.1371%2Fjournal.pone.0018410)[4](https://www.ncbi.nlm.nih.gov/pmc/articles/PMC8068577/). This makes it a practical choice for wound healing research. 4. The search results indicate that automated image analysis techniques, such as convolutional neural networks, can be used to quantify re-epithelialization and other wound healing parameters from H&E stained tissue sections[4](https://www.ncbi.nlm.nih.gov/pmc/articles/PMC8068577/). This enables more objective, high-throughput analysis.   In summary, the key advantages of using H&E staining to measure wound re-epithelialization are the ability to directly visualize and quantify this process, the comprehensive view of wound healing it provides, the technical simplicity and cost-effectiveness of the method, and the potential for automated quantitative  Additionally, the majority of wound papers just use h&e to measure wound re-epithelialization. Below are 4 recent ones from the most highly ranked skin biology jo9urnal JID, and 3 from this journal PLoS One, that use h&E.  1: Xu H, Yang H, Wang Z, Tang Q, Cao X, Chen C, Dong Y, Xu Z, Lv D, Rong Y, Chen M, Tang B, Deng W, Zhu J, Hu Z. Epidermal Stem Cell Derived Exosomes Alleviate Excessive Autophagy Induced Endothelial Cell Apoptosis by Delivering miR200b-3p to Diabetic Wounds. J Invest Dermatol. 2023 Oct 13:S0022-202X(23)02951-2. doi: 10.1016/j.jid.2023.08.030. Epub ahead of print. PMID: 37838331.  2: Kida M, Fatima I, Rozhkova E, Otero-Viñas M, Wu M, Kalin JH, Cole PA, Falanga V, Alani RM, Sharov AA. Inhibition of the CoREST Repressor Complex Promotes Wound Re-Epithelialization through the Regulation of Keratinocyte Migration. J Invest Dermatol. 2024 Feb;144(2):378-386.e2. doi: 10.1016/j.jid.2023.07.022. Epub 2023 Aug 25. PMID: 37633457; PMCID: PMC10790709.  3: Roy R, Mahmud F, Zayas J, Kuzel TM, Reiser J, Shafikhani SH. Reduced  Bioactive Microbial Products (Pathogen-Associated Molecular Patterns) Contribute to Dysregulated Immune Responses and Impaired Healing in Infected Wounds in Mice with Diabetes. J Invest Dermatol. 2024 Feb;144(2):387-397.e11. doi: 10.1016/j.jid.2023.08.004. Epub 2023 Aug 22. PMID: 37619833; PMCID: PMC10840742.  4: Lu JY, Wang XQ, Fu ZB, Gao LH, Mannam H, Xiang YP, Joo YY, Zeng JR, Wang D, Paller AS. Topical Ozone Accelerates Diabetic Wound Healing by Promoting Re-Epithelialization through the Activation of IGF1R-EGFR Signaling. J Invest Dermatol. 2023 Dec;143(12):2507-2514.e6. doi: 10.1016/j.jid.2023.05.015. Epub 2023 Jun 8. PMID: 37295490.  5: Iswara A, Tanaka K, Ishijima T, Nakajima Y, Mukai K, Tanaka Y, Nakano Y,  Sugama J, Oe M, Okuwa M, Nakatani T. Wound healing in db/db mice with type 2 diabetes using non-contact exposure with an argon non-thermal atmospheric pressure plasma jet device. PLoS One. 2022 Oct 14;17(10):e0275602. doi:10.1371/journal.pone.0275602. PMID: 36240146; PMCID: PMC9565687.  6: Mukai K, Horike SI, Meguro-Horike M, Nakajima Y, Iswara A, Nakatani T.  Topical estrogen application promotes cutaneous wound healing in db/db female mice with type 2 diabetes. PLoS One. 2022 Mar 10;17(3):e0264572. doi:10.1371/journal.pone.0264572. PMID: 35271602; PMCID: PMC8912242.  7: Moreira KG, do Prado TP, Mendes NF, de Medeiros Bezerra R, Jara CP, Melo Lima MH, de Araujo EP. Accelerative action of topical piperonylic acid on mice full thickness wound by modulating inflammation and collagen deposition. PLoS One. 2021 Oct 26;16(10):e0259134. doi: 10.1371/journal.pone.0259134. PMID: 34699564; PMCID: PMC8547657. |

| 2 | Comment | Authors should show the other markers of wound healing such as pro-inflammatory cytokines (IL1, TNF-alpha, etc.) either as immunohistochemistry or RNA or Western analysis of the skin samples. |
| --- | --- | --- |
|  | Response | We agree with the reviewer that it would be good to show other markers, but at the 3^rd^ round of review and after almost 12 months, we simply do not have the resources, nor the personnel to do so. We do then acknowledge the limitations of this study in the conclusions. Every research project has limitations, and research is never done (it’s good otherwise we will be out of a job). We do believe strongly, however, that federally funded research needs to be shared with the community and we believe the current work, even with these limitations, meets the standards for publications in PLOS One.  “These are preliminary results and additional future studies using keratin 14 staining to evaluate keratinocites as well as other markers of wound healing such as pro-inflammatory cytokines either as immunohistochemistry or RNA or Western analysis of the skin samples will be beneficial to confirm the conclusions of this work.  “ |

| 3 | Comment | Figure 3, Panel B: It is not clear as what is on the right panel (I assume it is the zoom image of the left edge and right edge of the wound). This description can be in the legends. Also, on the right panel, it will be better if there is heading on the top (like, Left edge or Right Edge. |
| --- | --- | --- |
|  | Response | We agree with the reviewer that our figure needs improvement. Figure 3B was updated to be more specific for the audience. Please see update bellow:   |

| 4 | Comment | Figure 3 C and D: Please clearly state if the data presented is shown in SEM or SD for each of the panel. It is also better if authors can stick to SEM or SD in all the figures in this manuscript. Also, correct this information in the Methods for Statistical Analysis section. |
| --- | --- | --- |
|  | Response | Plots in figure 3 C and D both show SEM; this is now mentioned in the figure legend. The Methods section has been updated to better describe the statistical analysis which was performed." |

| 5 | Comment | Figure 3, panel A: Is there a reason behind keeping the iNOS alone, CD206 alone, F4/80 alone, and DAPI alone staining in the upper two rows in black and white? Why not a colored one? |
| --- | --- | --- |
|  | Response | We have updated the figure to contain colored images of iNOS, CD206, F4/80, and DAPI. All images have been labeled accordingly with pseudo colors. Please see updated image below:   |

| 6 | Comment | Abstract, Line 26: It will be better to write “an in vivo mouse model” instead of “a mouse model in vivo”. |
| --- | --- | --- |
|  | Response | We agree with the reviewer’s request, and we have updated the sentence accordingly. |

| 7 | Comment | Results, Line 199: Please make it clear in the methods that both the wounds on single mouse received the same treatment (control or EF). |
| --- | --- | --- |
|  | Response | We agree with the reviewer and made the appropriate edits that make it clear that both the wounds on the mouse received the same treatment (control or EF).  I incorporated the following sentence, " Both the wounds on the mouse received the same treatment, either control or EF”, in line 202-203 where it made the most sense to make the clarification. |
